# Multimodal AI-based modeling of glaucoma progression: a 3PM-guided approach integrating structural, functional, and vascular patterns

**DOI:** 10.1007/s13167-025-00429-0

**Published:** 2025-11-06

**Authors:** Natalia I. Kurysheva, Oxana Ye. Rodionova, Alexey L. Pomerantsev, Saina I. Ponomareva, Olga Golubnitschaja

**Affiliations:** 1 The Ophthalmological Center of the Federal Medical and Biological Agency of the Russian Federation, 15 Gamalei Street, Moscow, 123098 Russian Federation; 2 Federal Research Center for Chemical Physics RAS, 4, Kosygin Street, Moscow, 119991 Russian Federation; 3 Predictive, Preventive and Personalised (3P) Medicine, University Hospital Bonn, Rheinische Friedrich-Wilhelms-Universität Bonn, 53127 Bonn, Germany

**Keywords:** Predictive preventive personalized medicine (PPPM / 3PM), Glaucoma progression, Disease modeling, Primary open-angle glaucoma, OCT-angiography, Individualized protection against irreversible blindness, Patient phenotyping and stratification, Multi-modal risk assessment, Biomarker panels, Application of AI in medicine, Machine learning, Improved individual outcomes

## Abstract

**Background:**

Glaucoma remains the leading cause of irreversible blindness worldwide. The development of predictive, preventive, and personalized medicine (3PM) strategies in the area is essential to address high inter-individual heterogeneity in glaucoma progression, in order to effectively protect stratified patients against disease progression.

**Aim:**

This study aims to develop and validate a personalized, multimodal predictive modeling framework that integrates structural, functional, and vascular biomarkers for individualized risk stratification of progression rates in primary open-angle glaucoma (POAG).

**Methods:**

Patients with POAG at varying stages were monitored for at least 36 months and underwent comprehensive multimodal evaluation, including structural optical coherence tomography (OCT), OCT angiography (OCT-A), automated perimetry, and biomechanical assessments. Predictive modeling was performed using Ranked Partial Least Squares Discriminant Analysis (Ranked PLS-DA). Model performance and variable importance were established through Procrustes Cross-Validation and optimization procedures.

**Results and data interpretation in the framework of 3PM:**

The final models included up to 27 parameters in early-stage POAG and 20 in advanced disease, leading to high prognostic accuracy (AUC up to 0.90) for classifying slow, moderate, and rapid rates of glaucoma progression. Feature importance analysis demonstrated that different biomarkers dominate at different disease stages: RNFL thickness, peripapillary microvascular dropout, parafoveal vascular density and corneal hysteresis in early POAG, while age, ganglion cell complex thickness, specific macular thickness measures, and peripapillary perfusion parameters were most predictive in advanced stages.

**Conclusions and 3PM-relevant outlook:**

The proposed innovation utilizes multimodal predictive disease modeling that supports accurate risk stratification, personalized glaucoma management and individualized protection against disease progression. Successful clinical application requires initial profiling, regular model recalibration, and adaptive treatment strategies – altogether leading to improved visual outcomes in stratified patients and leveraging resources used.

## Background

### Global impact and societal burden of glaucoma

Glaucoma is the leading cause of irreversible blindness worldwide, disproportionately affecting aging populations and presenting significant global and regional health challenges. The estimated global prevalence of glaucoma among individuals aged 40–80 years is 3.54%, with primary open-angle glaucoma (POAG) accounting for 3.05% (95% credible interval: 1.69–5.27), which means that POAG comprises approximately 86% of all glaucoma cases in this age group. By 2020, the number of people (aged 40–80 years) living with glaucoma globally was projected to reach 76.0 million, with further growth to 111.8 million expected by 2040, driven largely by population aging and demographic transitions [1]. More than half of affected individuals remain undiagnosed, particularly in lower-income countries, which often leads to late-stage presentation and permanent vision loss [2].

The societal and economic burden of glaucoma is immense across nations. In the United States, annual expenditures for glaucoma management approach $5.8 billion and are projected to reach $17.3 billion by 2050 [3]; in Europe, yearly therapy costs may range from €153 to €1,194 per patient [4]; and in developing countries, glaucoma treatment can consume up to 5% of a household’s annual income, severely limiting access to consistent therapy [5]. Patients with glaucoma face not only direct expenses related to treatment, but also substantial indirect costs due to reduced productivity, work absences, risk of injuries (such as falls), and long-term vision loss. Quality of life is fundamentally threatened: patients experience limitations in reading, walking, driving, and social participation, and glaucoma is a known risk factor for depression and social isolation [6]. Ultimately, glaucoma’s growing prevalence and persistent underdiagnosis produce a profound and multifaceted burden for patients, families, healthcare systems, and societies worldwide, underscoring the critical importance of early detection, individualized monitoring of disease progression and effective interventions.

The centrality of predictive, preventive and personalized medicine (PPPM) for addressing these challenges and creating advanced strategies in ophthalmology has been comprehensively outlined in the EPMA position paper, which established PPPM as a fundamental framework for translational medical research and clinical care development [7].

### Clinical heterogeneity in glaucoma progression rates

Clinical heterogeneity in glaucoma progression poses a major challenge, as patients with similar baseline findings can experience vastly different disease courses. Average mean deviation (MD) decline (a measure of overall visual field sensitivity loss) in treated eyes ranges from − 0.05 to −0.62 dB/year across studies, while progression rates exceeding − 1.5 dB/year, affecting 3–17% of patients, are considered problematic and require aggressive management [8]. Advanced glaucoma cohorts show MD rates of −0.67 ± 0.49 dB/year in nearly half of patients despite therapy [9]. The heterogeneity becomes even more pronounced when examining specific patient subgroups and disease subtypes. Analysis of 118 untreated patients from the Early Manifest Glaucoma Trial followed for at least 6 years revealed striking differences among glaucoma subtypes: while the mean overall progression rate was − 1.08 dB per year, high-tension glaucoma progressed at −1.31 dB/year, normal-tension glaucoma at −0.36 dB/year, and pseudoexfoliation glaucoma at −3.13 dB/year, representing dramatically accelerated deterioration [10]. Age also significantly influences progression variability, with older patients demonstrating markedly greater susceptibility to retinal nerve fiber layer loss. For example, at an intraocular pressure of 24 mmHg, a 40-year-old patient would have retinal nerve fiber layer loss of −0.78 μm/year, but an 80-year-old patient would progress at −1.34 μm/year, representing 72% faster deterioration [11].

Numerous risk factors contribute to this heterogeneity: elevated intraocular pressure (IOP) and larger diurnal IOP fluctuations, thin central corneal thickness, greater baseline visual field loss, optic disc hemorrhages, reduced ocular perfusion pressure and systemic vascular dysregulation, older age, cardiovascular and cerebrovascular comorbidities, and adherence issues all increase progression risk [12, 13]. However, even in the absence of these established risk factors, some patients may still experience rapid deterioration [14]. This underscores the limitations of conventional predictors and highlights the inherent unpredictability of glaucoma progression, emphasizing the need for more comprehensive and individualized approaches to risk assessment and patient management.

## The role of machine learning in glaucoma prognostics

Machine learning (ML) has revolutionized glaucoma prognostics by enabling multimodal progression assessment and patient-specific risk estimation. Leveraging large datasets - visual field series, three-dimensional OCT scans, and detailed clinical profiles. ML algorithms detect complex, nonlinear progression signatures beyond the reach of traditional linear models. Shuldiner et al. demonstrated that ML algorithms can predict rapid glaucoma progression (defined as MD decline > 1.0 dB/year) based solely on initial visual field testing, with their support vector machine model achieving an AUC of 0.72 using baseline visual field parameters, reliability metrics, and patient age [15]. Dixit et al. trained a convolutional long short‑term memory (LSTM) on longitudinal visual field sequences augmented with baseline cup-to-disc ratio, central corneal thickness, and IOP, improving progression detection from an AUC of 0.79–0.82 (visual field data alone) to 0.89–0.93 when clinical parameters were included [16]. Hussain et al. synthesized future OCT B-scans using a conditional generative adversarial network (GAN) and combined them with real OCT volumes, visual field mean deviations, intraocular pressure measurements, and demographic data in a convolutional neural network (CNN)–LSTM framework, achieving an AUC of 0.83 for predicting a ≥ 3 dB MD decline at 12 months and 0.81 when forecasting 9 months prior to functional loss [17]. Notably, across these studies, AUC consistently increases as additional data modalities and timepoints are incorporated, underscoring the value of enriched, multimodal inputs for enhancing prognostic accuracy.

## Working hypothesis in the framework of 3P medicine

The present study addresses a critical gap in glaucoma research: the absence of multimodal, stage-specific predictive models that integrate optical coherence tomography angiography (OCT-A) with conventional structural and functional biomarkers. We hypothesize that integrating OCT-A metrics: retinal and peripapillary vessel density and choroidal parameters with conventional biomarkers may improve predictive accuracy in overall glaucoma management.

For achieving the goal, we proposed to include retinal nerve fiber layer (RNFL) and ganglion cell complex (GCC) thickness, lamina cribrosa curvature indices (LCCI), perimetric parameters (Visual Field Index -VFI, Mean Deviation - MD, Pattern Standard Deviation - PSD), and to create separate Ranked Partial Least Squares Discriminant Analysis (Ranked PLS-DA) models for early-stage and advanced POAG cohorts. This approach was expected to enable precise, individualized stratification of glaucoma progression rates. The porposed inclusion of OCT-A was expected to provide unique insights into microvascular alterations that precede and predict structural and functional decline.

The proposed innovation is expected to significantly contribute to the paradigm change from reactive to proactive glaucoma management, namely.

- by enabling early identification of high-risk patients through vascular and structural biomarkers – the predictive component of the proposed innovation,
- guiding timely initiation of targeted surveillance and prophylactic therapies before irreversible damage occurs – the preventive component of 3PM approach, and
- customizing follow-up schedules, pharmacologic regimens, and surgical intervention thresholds tailored to indvidiualized patient profiles following the concept of personalization of treatments in the 3PM strategy.

## Study design

### Clinical approach

The study was performed in accordance with the ethical principles specified in the Declaration of Helsinki, the Good Clinical Practice (GCP), and regulatory requirements. The study included 256 eyes from 256 patients with POAG at early, moderate, advanced stages. All patients were managed at the Burnazyan Federal Biophysical Center and Ophthalmological Center in Moscow between January 2019 and December 2023, with each eye observed for at least 36 months.

Inclusion criteria comprised a diagnosis of POAG at early, moderate, or advanced stages based on the presence of elevated intraocular pressure (IOP), neuroretinal rim thinning, and focal or diffuse RNFL defects with corresponding visual field changes [18]. All study participants additionally had a spherical equivalent between − 6.0 D and + 6.0 D, astigmatism ≤ 2.0 D, and an open anterior chamber angle ≥ 30° on gonioscopy.

Exclusion criteria encompassed any media opacities precluding reliable OCT or visual field testing (for example, dense cataract or corneal scar), as well as unreliable visual field examinations (defined by fixation losses > 20% or false positives/negatives > 15%), fewer than five reliable visual field tests, prior intraocular surgery (including cataract extraction, trabeculectomy, or laser trabeculoplasty), systemic disorders affecting ocular perfusion or neural function (such as diabetes mellitus, systemic autoimmune disease, Parkinson’s disease, Alzheimer’s disease, or dementia), and the use of systemic miotics or other medications known to alter pupil size.

Patients meeting the inclusion and exclusion criteria were stratified by disease stage. The first cohort comprised from patients with early-stage POAG, while the second cohort comprised eyes from patients with moderate and advanced stages of the disease. All patients underwent a standardized ophthalmic evaluation at each visit, including medical history review, visual acuity testing, autorefraction, slit-lamp biomicroscopy, ophthalmoscopy, gonioscopy, IOP measurement using the Ocular Response Analyzer (ORA; Reichert, USA), pachymetry (SP-100; Tomey, Germany), and standard automated perimetry with the SITA Standard 24 − 2 protocol (Humphrey Field Analyzer; Carl Zeiss Meditec, USA). Spectral-domain OCT and OCT angiography were performed using the RTVue XR Avanti device with AngioVue OCTA capability (Optovue, Inc., USA).

Glaucomatous optic neuropathy progression rates were determined from both perimetric and OCT data. Guided Progression Analysis (GPA) software on the Humphrey Field Analyzer II was used to assess visual field progression by trend analysis of the VFI and by event analysis. Progression was considered statistically significant when the slope of the 24 − 2 VFI trend had *p* < 0.05. Only reliably flagged test points were included in the calculation of mean progression rates. Standard automated perimetry was performed every 6 months. A study eye was classified as “progressing” when either the event analysis or the trend analysis indicated significant progression. To eliminate confounding by cataract, eyes with documented cataract progression - defined as a decrease of two or more lines of visual acuity on at least two visits due to lens opacity - were excluded from perimetric progression analysis.

Changes in RNFL and GCC thickness were analyzed using the built-in trend analysis software of the RTVue XR Avanti OCT (Optovue, Inc.). Three consecutive scans were acquired at each visit, and only scans with a signal strength index (SSI) > 45 were included. Structural progression was defined by a statistically significant negative slope (*p* < 0.05) of the regression line for RNFL or GCC thickness over time.

At the end of follow-up, each patient received an expert-graded progression category based on these criteria: (1) slow progression, defined by a rate of progression (ROP) in VFI of 0.5–1.0% per year and ROP in RNFL and GCC thickness of < 1 μm/year; (2) moderate progression, defined by ROP in VFI of 1.0–2.0% per year and ROP in RNFL and GCC of 1–2 μm/year; and (3) rapid progression, defined by ROP in VFI > 2.0% per year and ROP in RNFL and GCC > 2 μm/year [18, 19].

### Machine learning approach

For building the prognostic model of glaucomatous optic neuropathy (GON) progression rate, we employed Ranked Partial Least Squares Discriminant Analysis (Ranked PLS-DA) - an innovative variation of PLS-DA that accounts for class ranking and implements soft discrimination in multiclass classification [20].

The application of Ranked PLS-DA is particularly relevant for the cases involving small patient cohorts, complex multicollinearity, and the need for model interpretability. This method has been demonstrated to possess several advantages over conventional approaches - it takes into account the predictor redundancy and correlation, while enabling correct selection of the most significant variables influencing disease outcomes. Ranked PLS-DA is especially effective when class hierarchy exists or smooth transitions occur between severity levels or progression rates, which is typical for ophthalmological applications.

The fundamental principle of Ranked PLS-DA is its ability to model ordinal relationships between classes while maintaining the flexibility of soft classification. Unlike traditional hard classification methods that unambiguously assign samples to the predefined categories, this approach recognizes that disease progression exists on a continuum, allowing for probabilistic assignments that better reflect clinical reality. This is particularly valuable in glaucoma research, where the boundaries between slow, moderate, and rapid rates of progression are indistinct.

The method develops a projection onto the latent space that maximize the separation between ordered classes while preserving the underlying data structure. This dual optimization ensures both high discriminatory power and biological relevance of the resulting model. The soft discrimination capability allows identification of borderline cases that may require enhanced monitoring or individualized treatment approaches.

Variable selection was performed using a novel wrapper method [21], based on the sequential removal of variables from the model. Variables were retained if their removal significantly decreased model performance, ensuring that only the most predictive parameters were included in the final model.

For model optimization and validation, an augmented test set was generated using Procrustes Cross-Validation (PCV) [22]. This method creates a validation set that can be utilized analogously to an independent test set, providing reliable performance assessment while allowing the use of limited clinical data. The PCV approach is particularly valuable in medical applications where large datasets are challenging to obtain, as it provides consistent validation metrics without requiring an additional cohort of patients.

Model performance was evaluated using multiple complementary metrics including sensitivity, specificity, total efficiency (TEFF), and area under the ROC curve (AUC) [23]. This comprehensive evaluation framework ensures robust assessment of the model’s predictive capabilities across different performance dimensions, providing clinicians with confidence in the model’s sustainability for real-world applications.

## Results and data interpretation

### Patient data analysis

The final cohort included 114 patients (114 eyes) who met the inclusion criteria. The first group of patients with early-stage POAG included 59 patients, while the second group with advanced and severe stages of POAG comprised 55 patients. The clinical characteristics of the patients are presented in Table 1.

During the 36-month observation period, progression rates varied significantly within each group. In the early-stage cohort, slow rate of progression was documented in 21 patients, moderate rate of progression in 18 patients, and rapid rate of progression in 20 patients. The advanced/severe stage cohort showed a different distribution pattern, with slow rate of progression observed in 14 patients, moderate rate of progression in 18 patients, and rapid rate of progression in 23 patients.

**Table 1:** Clinical characteristics of patients at baseline

Parameter	Abbreviation	Group 1 (Early stage)	Group 2 (Moderate/Advanced Stages)	*p* value
Age, years	Age	68.31 ± 9.85	73.65 ± 8.01	0.002*
Gender, male/female, abs.	Gend	46/13	24/31	0.286
Corneal hysteresis, mmHg	CH	10.12 ± 1.80	10.14 ± 2.25	0.952
Intraocular pressure, mmHg	IOP	19.55 ± 4.80	17.61 ± 4.52	0.029*
Spherical equivalent, diopters	SE	−0.83 ± 1.79	−1.20 ± 1.84	0.270
Central corneal thickness, µm	CCT	550 ± 19.12	536.20 ± 28.23	0.003*
Mean deviation index of retinal light sensitivity, dB	MD	−1.81 ± 2.31	−14.05 ± 7.02	< 0.001*
Pattern standard deviation index of retinal light sensitivity, dB	PSD	2.24 ± 1.13	9.17 ± 3.28	< 0.001*
Peripapillary Retinal Nerve Fiber Layer thickness, µm	RNFL_ave	82.86 ± 12.26	66.02 ± 12.45	< 0.001*
RNFL thickness in superotemporal sector, µm	RNFL_ST	106.14 ± 22.04	80.11 ± 17.68	< 0.001*
RNFL thickness in superonasal sector, µm	RNFL_SN	84.36 ± 14.96	72.80 ± 21.04	< 0.001*
RNFL thickness in nasal-upper sector, µm	RNFY_NU	73.42 ± 11.52	60.45 ± 14.09	< 0.001*
RNFL thickness in nasal-lower sector, µm	RNFL_NL	68.15 ± 13.03	55.47 ± 13.57	< 0.001*
RNFL thickness in inferonasal sector, µm	RNFL_IN	85.07 ± 17.38	71.60 ± 16.48	< 0.001*
RNFL thickness in inferotemporal sector, µm	RNFL_IT	102.68 ± 26.03	77.38 ± 23.42	< 0.001*
RNFL thickness in temporal-lower sector, µm	RNFL_TL	64.81 ± 14.12	52.31 ± 22.85	< 0.001*
RNFL thickness in temporal-upper sector, µm	RNFL_TU	74.07 ± 14.54	55.47 ± 14.84	< 0.001*
Ganglion cell complex thickness, µm	GCC	87.31 ± 11.62	68.96 ± 10.60	< 0.001*
Vascular density in parafovea superior, %	Parafovea_VD_S	40.68 ± 5.95	37.13 ± 6.34	0.003*
Vascular density in parafovea inferior, %	Parafovea_VD_I	40.22 ± 5.48	37.65 ± 6.50	0.024*
Radial peripapillary capillary vascular density superior, %	VD_Disc_S	45.83 ± 7.10	34.64 ± 8.94	< 0.001*
Radial peripapillary capillary vascular density inferior, %	VD_Disc_I	43.99 ± 7.52	34.54 ± 8.54	< 0.001*
Parafoveal retinal thickness superior, µm	Parafovea_Th_S	312.69 ± 18.76	287.07 ± 36.55	< 0.001*
Parafoveal retinal thickness inferior, µm	Parafovea_Th_I	311.19 ± 17.81	284.49 ± 35.70	< 0.001*
Average superficial macular vascular density, %	wiVD_Superficial	40.53 ± 5.12	33.95 ± 5.89	< 0.001*
Average deep macular vascular density, %	wiVD_Deep	41.92 ± 4.77	37.26 ± 4.92	< 0.001*
Mean retinal thickness, µm	wi_Th	272.63 ± 15.02	250.95 ± 21.20	< 0.001*
β-zone peripapillary choroidal atrophy area, mm²	β-PPA	1.28 ± 0.98	1.31 ± 1.09	0.874
Area of choriocapillaris dropout, mm²	MvD_area	0.07 ± 0.06	0.28 ± 0.17	< 0.001*
Angular circumferential extent of choriocapillaris dropout, °	Angle	29.76 ± 11.78	63.24 ± 20.07	< 0.001*
Choroidal thickness at fovea, µm	Foveal_CT	296.11 ± 18.03	218.73 ± 60.19	< 0.001*
Peripapillary choroidal thickness, µm	Peripapillary_CT	177.29 ± 6.21	142.78 ± 49.43	< 0.001*
Lamina cribrosa thickness, µm	LCT	258.85 ± 9.89	214.93 ± 31.09	< 0.001*
Lamina cribrosa curvature index	LCCI	7.79 ± 0.75	12.45 ± 2.63	< 0.001*

*Statistically significant results are marked with an asterisk.

For building the initial model for each group, 34 variables presented in Table 1 were used. After optimization procedures and variable selection, the number of variables was reduced to 27 for Group 1 and to 20 for Group 2. Model characteristics are presented in Table 2.

**Table 2:** Ranked PLS-DA model performance metrics

Patient Group	Model Type	Total Specificity (TSPC)	Total Sensitivity (TSNS)	Total Efficiency Training Set (TEFF train)	Total Efficiency Test Set (TEFF test)	Area Under ROC Curve (AUC)
Group 1 (Early stage)	Initial model (34 variables)	0.78	0.82	0.80	0.76	0.90
Group 1 (Early stage)	Optimized model (27 variables)	0.86	0.81	0.83	0.77	0.90
Group 2 (Advanced/severe stages)	Initial model (34 variables)	0.76	0.78	0.77	0.72	0.86
Group 2 (Advanced/severe stages)	Optimized model (20 variables)	0.78	0.93	0.85	0.77	0.90

**Fig. 1:**
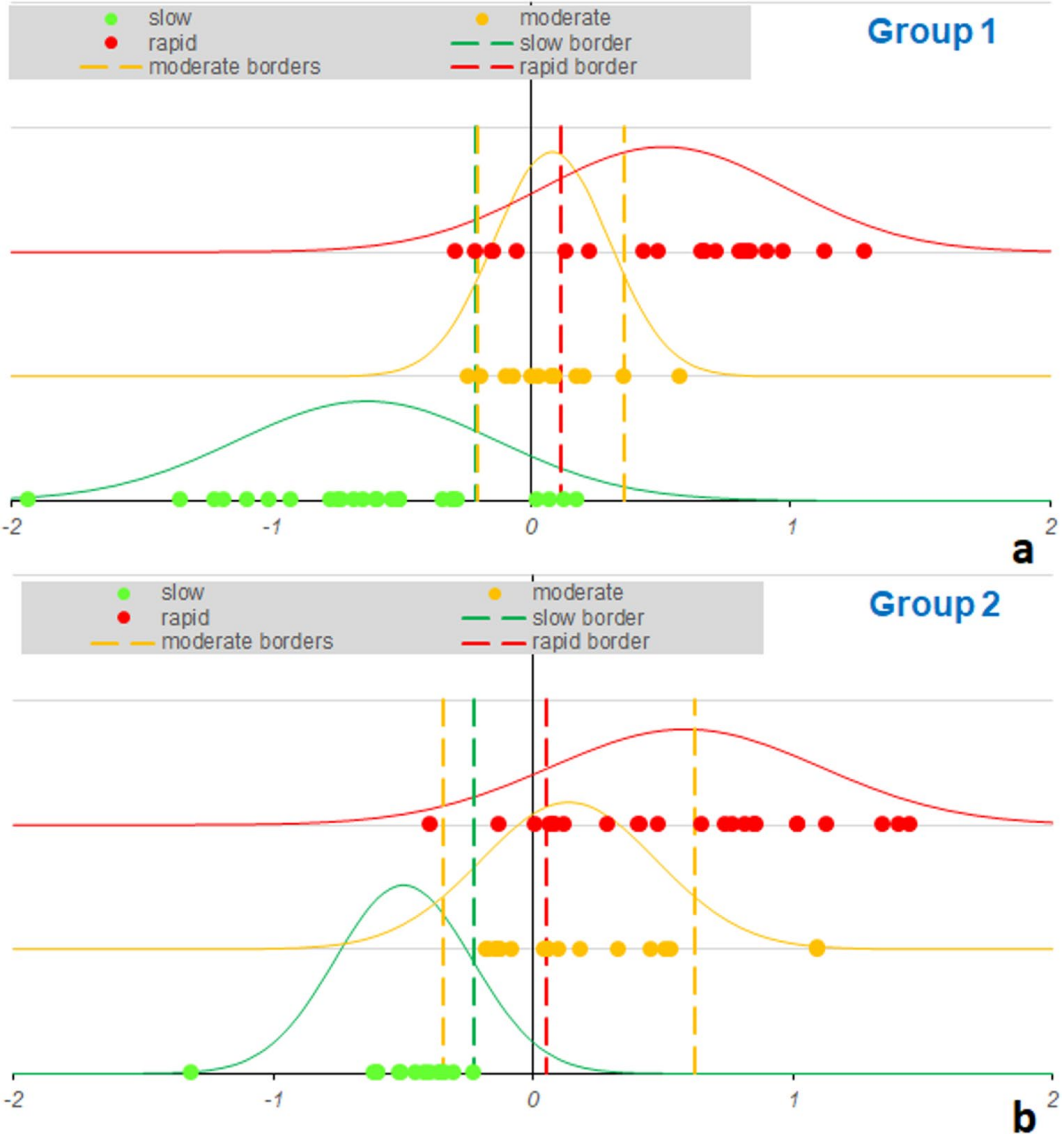
Probabilistic distributions of predicted values for patients by progression rate. a – Group 1, b – Group 2; Color code: green - slow, yellow - moderate, red - rapid. Vertical lines mark class boundaries; “borderline” zones near these lines represent overlap, and points here may require additional clinical review

The results of Ranked PLS-DA modeling are presented in Fig. 1. This figure visualizes the probabilistic distributions of predicted values for the slow, moderate, and rapid rates of glaucoma progression in the optimized model.

The discriminating threshold values (boundaries) between classes define so-called “critical zones” where misclassification is possible, primarily between adjacent classes. A significant portion of overlaps is observed at the boundary between the moderate and rapid rates of progression, as well as between the moderate and slow progression, which reflects natural clinical heterogeneity and the continuous nature of visual function loss. The rightmost distribution (rapid rate of progression) exhibits the most pronounced “separation” from other classes, indicating a more defined phenotype of patients with marked and rapid rates of functional loss.

Figure 1 highlights points corresponding to patients whose classification is questionable: some samples fall into “borderline” zones and can be considered as “targets” for expert opinion revision and more intensive monitoring. This approach reduces the risks of incorrect stratification and enhances the clinical significance of the model.

### Key predictive factors by disease stage

The variable importance analysis revealed distinct stage-specific predictor patterns that reflect the evolving pathophysiology of glaucomatous progression. The predictive models demonstrated fundamentally different priorities between early-stage and advanced-stage disease, with structural parameters dominating in early detection while a more diverse set of biomarkers becomes critical in advanced stages.

#### Early-Stage glaucoma predictors

**Fig. 2:**
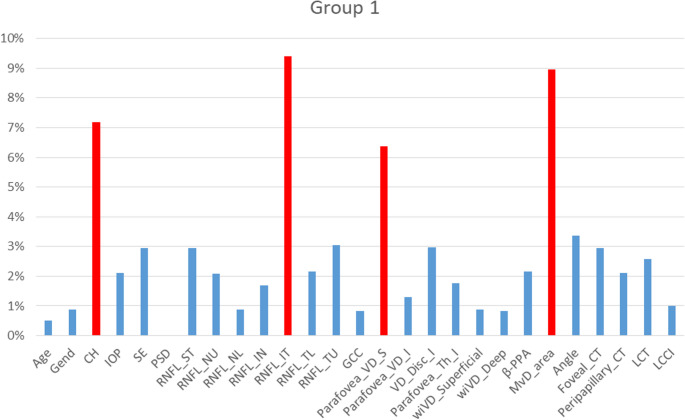
Comparative importance of predictors in the effectiveness of the prognostic model for Group 1. Red bars show the most significant predictors; values reflect VIP-scores by Ranked PLS-DA, with higher values indicating greater impact on progression risk stratification

In early-stage POAG, the optimized model identified retinal nerve fiber layer thickness in the inferotemporal sector (RNFL_IT) as the most critical predictor (Fig. 2). This finding emphasizes the vulnerability of the inferotemporal RNFL region in early glaucomatous damage, likely reflecting the anatomical susceptibility of nerve fibers entering the optic disc from this sector [24].

The microvascular dropout area (MvD_area) emerged as the second most important predictor (VIP-score 8.9), highlighting the central role of choriocapillaris perfusion deficits in early disease pathogenesis [25]. Corneal hysteresis (CH) ranked third (VIP-score 7.2), underscoring the importance of biomechanical corneal properties as a surrogate marker for scleral rigidity and optic nerve head susceptibility to pressure-induced damage [26].

Parafoveal vascular density superior (Parafovea_VD_S) demonstrated significant predictive value (VIP-score 6.4), supporting the hypothesis that macular vascular compromise occurs early in glaucomatous progression [27].

#### Advanced-stages of glaucoma

**Fig. 3:**
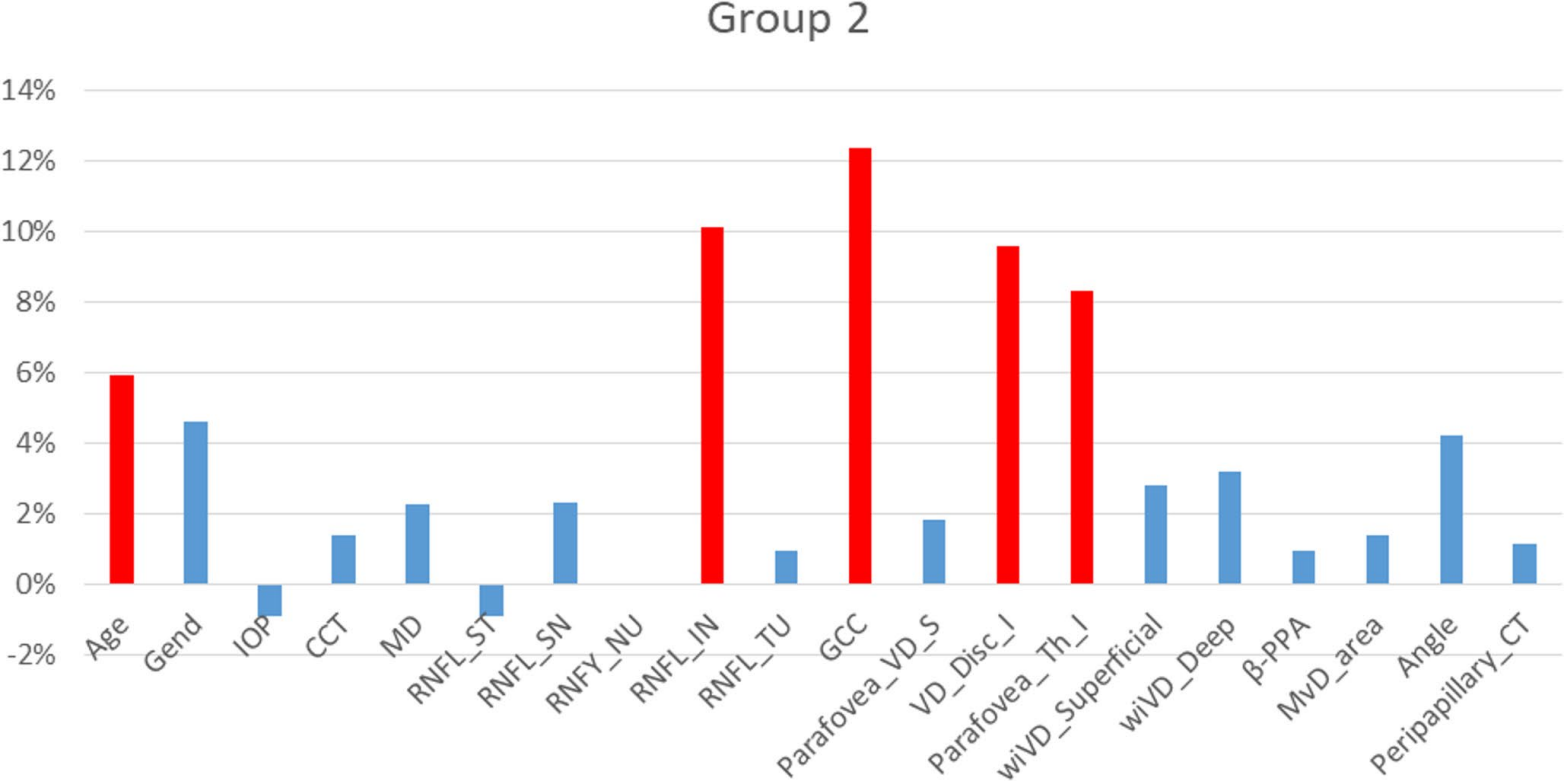
Comparative importance of predictors in the effectiveness of the prognostic model for Group 2. Red bars show the most significant predictors; values reflect VIP-scores by Ranked PLS-DA, with higher values indicating greater impact on progression risk stratification

The predictive landscape fundamentally shifted in advanced and severe glaucoma stages, where GCC emerged as the predominant predictor (see Fig. 3). This reflects the critical importance of macular ganglion cell integrity assessment in advanced disease, where residual functional capacity directly correlates with remaining retinal ganglion cells. Macular ganglion cells represent the final common pathway for central visual function, making their quantitative assessment directly relevant to remaining visual capacity. Unlike RNFL thickness, which can reach a measurement floor effect in advanced disease due to residual glial tissue and blood vessels, GCC thickness maintains its discriminatory power across all disease stages, providing a more accurate reflection of neuronal loss in severely damaged eyes [28]. This structural assessment framework is further enhanced by regional macular analysis, where parafoveal retinal thickness inferior (Parafovea_Th_I) contributed significantly (VIP-score 8.3), capturing localized damage patterns that complement global GCC measurements.

The peripapillary vascular density inferior (VD_Disc_I) showed substantial importance (VIP-score 9.6), indicating that peripapillary perfusion becomes more predictively relevant than macular vasculature in advanced stages. This shift from macular to peripapillary vascular emphasis reflects fundamental changes in glaucomatous pathophysiology as disease severity progresses [29].

With age, the risk of rapid glaucomatous optic neuropathy progression increases substantially. This is confirmed by results from large cohort studies - elderly patients show faster rates of retinal nerve fiber loss compared to younger patients at similar IOP values [11].

Our findings emphasize that vascular factors remain significant predictors of glaucoma progression at both early and advanced disease stages. Whether represented by parafoveal vessel density in the macular region or by peripapillary vascular metrics, disturbances in ocular blood flow and microcirculation were identified as critical at all phases of the disease. The fact that impaired ocular blood flow emerges as a critical risk factor in glaucoma reflects multiple vascular mechanisms, including both arterial hypertension (since many elderly patients suffer from hypertension) and Flammer syndrome phenotype (FSP), which may act as independent or synergistic drivers of glaucomatous progression. Given that arterial hypertension commonly coexists with POAG in the elderly population, vascular impairments in our cohort likely involve multifactorial pathways. Regarding the Flammer syndrome phenotype [30], the patients exhibit impaired autoregulation of ocular blood flow which is manifested by instability of the local perfusion even when IOP is within the normal range. Notably, these patients are at increased risk for fluctuating visual field deficits and rapid progression despite “adequate” IOP control. This outcome is supported by long-term studies showing higher rates of diffuse field loss and vascular events in this patient cohort.

**Fig. 4:**
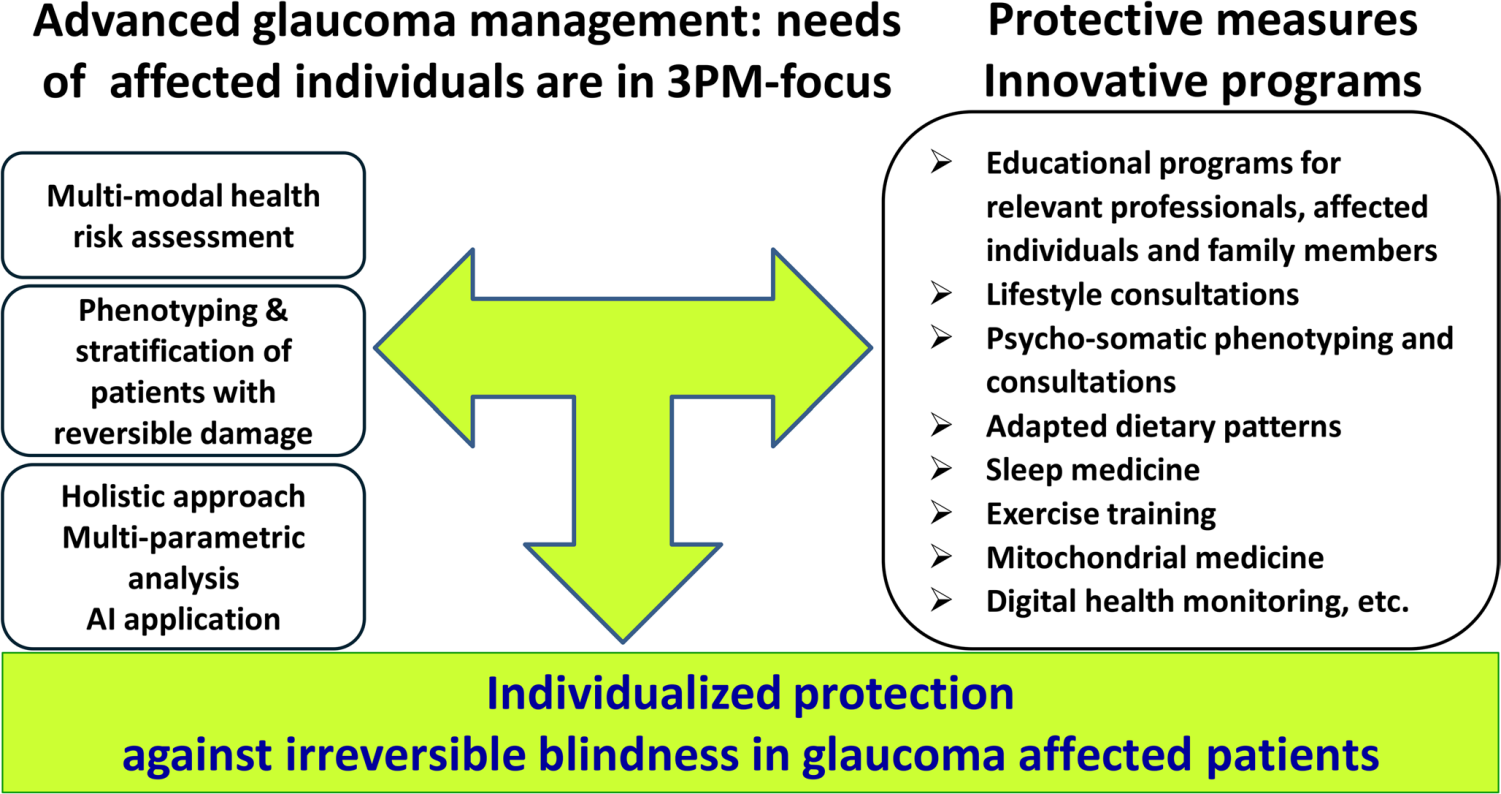
3PM-guided innovation to advance overall management of glaucoma: the paradigm change from reactive to proactive healthcare

Mounting research data demonstrate that FSP carriers are strongly predisposed to highly increased stress sensitivity [31]. Stress overload and increased sensitivity – both are associated with the FSP-specific psycho-somatic patterns and consequent epigenetic regulation which in turn is considered a target for vision restoration and recovery in glaucoma by individually adapted rehabilitation programs [32]. To this end, the FSP is characterized by chronication of the transient sympathoexcitation and its dominance over parasympathetic relaxation [33]. This sympathetic overdrive is associated with the cyclic ischemia-reperfusion potentially resulting in a progressive disease of small vessels, chronification of inflammation, and mitochondrial stress and burnout with a spectrum of associated pathologies in affected individuals [33]. FSP is usually manifested early in life being therefore instrumental for 3PM-guided individualised protection against health-to-disease transition in primary healthcare [33]. To meet needs of this patient cohort, comprehensive 3PM-guided protective measures are essential in primary and secondary care including individualized consultations towards appropriate life-style, dietary and physical activities linked to the mitochondrial health monitoring [34] as summarized in Fig. 4. To this end, patient friendly non-invasive approach utilising tear fluid multi-omics and mitochondria as vital biosensors has been established [35].

## Conclusions, expert recommendations, and outlook in the framework of 3PM

Multimodal predictive modeling of glaucoma progression, integrating structural (OCT), vascular (OCT-A), functional (perimetry), biomechanical, and demographic biomarkers, has demonstrated superior ability to stratify risk and forecast disease trajectories across all stages. Rather than relying on a few key metrics, our optimized models for early and advanced cohorts incorporate all measured predictors − 27 variables in early-stage and 20 in advanced-stage models to capture the full spectrum of disease heterogeneity. This comprehensive approach achieves high prognostic accuracy (AUC 0.90) and accommodates the continuous and multifaceted nature of glaucomatous damage.

To translate these findings into clinical practice changing the paradigm from reactive to proactive glaucoma management, we recommend below presented 3PM innovation:

### Predictive stratification

- Establish a baseline multimodal profile for every patient, encompassing structural, vascular, functional, biomechanical, and demographic data.
- Use the ML-assisted models to calculate individualized risk scores that guide surveillance intensity and intervention timing.

### Tailoring preventive measures

- Align follow-up intervals with predicted progression risk: more frequent monitoring (3–4 months) for high-risk profiles, extended intervals (9–12 months) for stable, low-risk eyes.
- Initiate prophylactic measures based on dominant risk domains (enhanced IOP control, vascular support, biomechanical optimization) before irreversible damage occurs.

### Personalized management plans

- Integrate predictive insights into treatment algorithms, customizing IOP targets, medication regimens, and surgical thresholds to each patient’s unique risk constellation.
- Reassess risk continuously at each visit, adapting management plans in real time as new multimodal data become available.

The 3PM-guided innovation is illustrated in Fig. 4.

## Data Availability

All the data used in this study are presented in this article.

## Abbreviations

PPPM/3PM: Predictive, Preventive, and Personalized Medicine
AI: Artificial Intelligence
ML: Machine Learning
GAN: Generative Adversarial Network
CNN: Convolutional Neural Network
LSTM: Long Short‑Term Memory
POAG: Primary Open‑Angle Glaucoma
GON: Glaucomatous Optic Neuropathy
OCT: Optical Coherence Tomography
OCT‑A: Optical Coherence Tomography Angiography
Ranked PLS‑DA: Ranked Partial Least Squares Discriminant Analysis
PCV: Procrustes Cross‑Validation
AUC: Area Under the ROC Curve
MD: Mean Deviation
PSD: Pattern Standard Deviation
VFI: Visual Field Index
RNFL: Retinal Nerve Fiber Layer
GCC: Ganglion Cell Complex
LCT: Lamina Cribrosa Thickness
LCCI: Lamina Cribrosa Curvature Index
CH: Corneal Hysteresis
IOP: Intraocular Pressure
CCT: Central Corneal Thickness
SE: Spherical Equivalent
VD: Vascular Density
wiVD_Superficial: Whole‑Image Superficial Vascular Density
wiVD_Deep: Whole‑Image Deep Vascular Density
wi_Th: Mean Whole‑Image Retinal Thickness
RNFL_ST: Retinal Nerve Fiber Layer, Superotemporal sector
RNFL_SN: Retinal Nerve Fiber Layer, Superonasal sector
RNFL_TU: Retinal Nerve Fiber Layer, Temporal‑Upper sector
RNFL_TL: Retinal Nerve Fiber Layer, Temporal‑Lower sector
RNFL_NU: Retinal Nerve Fiber Layer, Nasal‑Upper sector
RNFL_NL: Retinal Nerve Fiber Layer, Nasal‑Lower sector
RNFL_IN: Retinal Nerve Fiber Layer, Inferonasal sector
RNFL_IT: Retinal Nerve Fiber Layer, Inferotemporal sector
Parafovea_VD_S: Parafoveal Vascular Density, Superior
Parafovea_VD_I: Parafoveal Vascular Density, Inferior
VD_Disc_S: Radial Peripapillary Capillary Vascular Density, Superior
VD_Disc_I: Radial Peripapillary Capillary Vascular Density, Inferior
Parafovea_Th_S: Parafoveal Retinal Thickness, Superior
Parafovea_Th_I: Parafoveal Retinal Thickness, Inferior
β‑PPA: β‑zone Peripapillary Atrophy
MvD_area: Microvascular Dropout Area
Foveal_CT: Foveal Choroidal Thickness
Peripapillary_CT: Peripapillary Choroidal Thickness
Angle: Angular Extent of Choriocapillaris Dropout
ROP: Rate of Progression
TEFF: Total Efficiency
TSNS: Total Sensitivity
TSPC: Total Specificity
GPA: Guided Progression Analysis
GCP: Good Clinical Practice
FSP: Flammer Syndrome Phenotype
EPMA: European Association for Predictive, Preventive and Personalized Medicine
ORA: Ocular Response Analyzer
SSI: Signal Strength Index
